# Case 2/2017 - Cor Triatriatum, without Clinical Manifestation, in A 6-Year-Old Girl

**DOI:** 10.5935/abc.20170030

**Published:** 2017-03

**Authors:** Edmar Atik, Gláucia M. P. Tavares

**Affiliations:** Clínica Dr. Edmar Atik, São Paulo, SP - Brazil

**Keywords:** Cor Triatriatum, Congenital Abnormalities, Echocardiography, Signs and Symptoms

**Clinical data**: cardiac murmur was routinely identified at 2 years of age, which was characterized, on this occasion, as functional. A recent echocardiogram demonstrated a membranous partition in the left atrium with ample communication between the two cavities, proximal and distal. The child usually performs usual physical activities, just like other children.

**Physical examination**: eupneic, acyanotic, normal pulses. Weight: 19.6 kg; Height: 116 cm; Blood pressure: 95/60 mmHg; Heart rate: 78 bpm, oxygen saturation: 97%. Aorta not palpated in the suprasternal notch.

In the precordium, non-palpable *ictus cordis* and absence of systolic impulses in the Left Sternal Border (LSB). Normal heart sounds; systolic murmur, +/++/4, rough, medium LSB, variable in intensity, dependent on the position, sharply decreasing in the sitting position. The liver was not palpable.

## Complementary examinations

**Electrocardiogram** showed sinus rhythm and no signs of cavitary overload. The QRS complex had RS morphology in V1, qRs in V6. AP: +50°, AQRS: +80°, AT: +40°.

**Chest X-ray** showed normal cardiac area (cardiothoracic index: 0.46). The pulmonary vascular net was normal and the medial arch was rectified.

**Figure 1:**
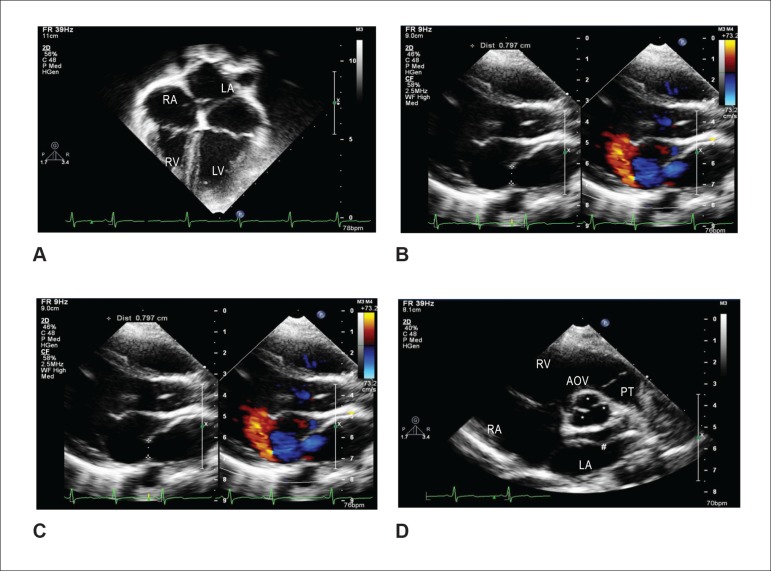
Echocardiograms demonstrate cor triatriatum in the 4-chamber apical plane in anatomical position, showing dilated left atrium, with septal defect (A); In the same plane, in black and white and with color mapping, showing the fenestration measurement and the flow passage in colors (B); in the long-axis parasternal plane, showing simultaneously, in black and white and with color mapping, the cor triatriatum membrane in the left atrium and the main fenestration measurement (C); and, in the short-axis parasternal plane, showing the septal defect in the left atrium (D). RA: right atrium; LA: left atrium; RV: right ventricle; LV: left ventricle; MV: mitral valve; AOV: aortic valve; TP: pulmonary trunk.

**Figure 2:**
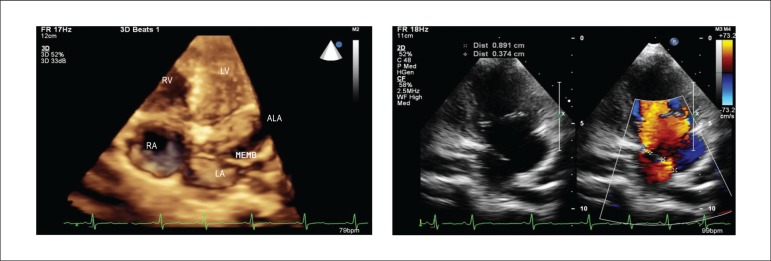
Echocardiograms in the 4-chamber apical plane in three-dimensional image acquisition, showing the left atrium divided by a fenestrated membrane and in the 2-chamber apical plane, simultaneously showing, in black and white and with color mapping, the cor triatriatum membrane in the left atrium, showing that there are two fenestrations, their measurements, and the flow passage in colors. RA: right atrium; LA: left atrium; MEMB: cor triatriatum membrane; RV: right ventricle; LV: left ventricle; ALA: accessory left atrium.

**Transthoracic echocardiogram** (Figures 1 and 2) showed a membrane in the middle of the left atrium, which was enlarged. The proximal cavity received the four pulmonary veins, and the distal cavity, in communication with the mitral valve, did not have any atrial septal defect. There were two fenestrations in the membrane, the largest being 10 mm and the smaller, 4 mm in diameter. The maximum transmembrane gradient was 9 mmHg, with a mean of 2.5 mmHg. The flow velocity through the pulmonary veins was normal and without turbulence, characterizing the absence of intra-atrial obstruction. The systolic pressure of the pulmonary artery was 30 mmHg. No other defects were found.

## Clinical diagnosis: *cor triatriatum sinister* with no associated defects (type A of the Lam classification) in an asymptomatic child, in the presence of mild intra-atrial flow limitation

**Clinical reasoning**: the available clinical elements were insufficient to characterize the existence of any congenital heart disease. The systolic murmur present in the LSB, variable and discrete, had functional characteristics, unrelated to any abnormality. Also, the usual complementary exams did not disclose any abnormalities. Thus, the left intra-atrial defect was discovered through a routine echocardiographic finding, motivated by the presence of functional murmur.

**Differential diagnosis**: other cardiac congenital anomalies can also be routinely diagnosed, without any suggestive elements, such as cardiac defects with mild effects, exteriorized by discrete and non-significant murmurs. Interatrial and interventricular septal defects, obstructive pulmonary and aortic valve lesions, coarctation of the aorta, and obstructions in the right ventricle and even in the left ventricle are examples of this situation.

**Conduct**: the ideal would be to remove the left intra-atrial membrane by surgical intervention. However, considering that this obstruction does not cause enough hemodynamic disorders to externalize any symptom or sign of a developing clinical problem, we chose the clinical observation until there is some manifestation.

**Comments**: *Cor triatriatum* is a rare anomaly, in which the atria are divided by a membrane, characterized as *sinister* to the left (present case) and *dexter* to the right.^1,2^ Embryology explains the anomaly by the inadequate incorporation of the pulmonary veins in the left atrium, causing the intra-atrial division. On the left side, the pulmonary veins drain into the proximal (posterosuperior) chamber, and the mitral valve and left atrial appendage are located in the distal (anteroinferior) chamber. *Cor triatriatum* is classified, according to Lam (1962), as type A, without associations (as in the present case); A1, in which the ASD occurs in the proximal chamber (50%); A2, in which the ASD occurs in the distal chamber (10%); B, in which the pulmonary veins drain into the coronary sinus (1%); and C, when there is Total Anomalous Pulmonary Venous Drainage (5%). Depending on the degree of the obstruction and associations, this obstructive anomaly can be diagnosed at any age.^1^ In the most severe situation, there is marked pulmonary venous drainage obstruction, pulmonary hypertension, and heart failure. Over the course of 50 years, 25 patients with *cor triatriatum* were operated at the Mayo Clinic, whose age ranged from 1 day to 73 years.^1^ The first corrective surgery for this anomaly was performed in 1956 by Lewis and, since then, approximately 250 cases have been surgically repaired. It is concluded that there is great diversity regarding the repercussion and also the age of clinical manifestation, and that it can be identified early in life and even in old age, when problems arise due to disease evolution, such as atrial fibrillation, pulmonary arterial hypertension and right heart failure.^2^ There are no reports of percutaneous involvement in this obstructive abnormality in the literature, but this idea is tempting, in view of the fact that this anomaly can be resolutively simpler, in the presence of an even more discrete residual obstruction.
